# Mapping the causal associations of cytokines with sarcopenia and aging traits: Evidence from bidirectional Mendelian randomization

**DOI:** 10.1002/jcsm.13456

**Published:** 2024-03-31

**Authors:** Mingchong Liu, Xiao Fu, Daqian Yu, Meng Li, Yutao Pan, Chensong Yang, Guixin Sun

**Affiliations:** ^1^ Department of Traumatic Surgery, Shanghai East Hospital, School of Medicine Tongji University Shanghai China

**Keywords:** aging traits, cytokines, growth factors, sarcopenia

## Abstract

**Background:**

Cytokines and growth factors may serve as a bridge in studying the causal relationships between inflammaging and sarcopenia due to their roles in inflammaging. In this study, we aim to explore the causal association of cytokines with sarcopenia and aging traits and further identify the significant inflammation factors.

**Methods:**

Bidirectional Mendelian randomization (MR) analysis was used to identify the causality. Forty‐one kinds of circulation cytokines and growth factors were set as exposures, and the data were from a summary genome‐wide association study (GWAS) containing three cohorts with 8293 healthy participants of European ancestry from 1983 to 2011. Hand grip strength, adjusted appendicular lean mass (AALM), usual walking pace, moderate‐to‐vigorous physical activity (MVPA) levels, able to walk or cycle unaided for 10 min (AWCU10) and telomere length were selected as outcomes. Data for outcomes were obtained from meta‐GWAS and the UK Biobank, and sample sizes ranged from 69 537 to 472 174. Low hand grip strength was defined by the European Working Group on Sarcopenia in Older People (EWGSOP) and Foundation for the National Institutes of Health (FNIH) cut‐off points, respectively. Other outcome traits were defined and measured according to the UK Biobank and raw cohorts' criteria. We set two significance thresholds for single nucleotide polymorphisms (SNPs) associated with exposures to obtain adequate SNPs (5 × 10^−6^ and 5 × 10^−8^). Inverse‐variance weighted, MR‐Egger and weighted median were employed to estimate the causality.

**Results:**

Twenty‐seven factors were identified to relate to sarcopenia and aging traits causally, and most were associated with only one outcome trait. IL16 (interleukin‐16), CTACK (cutaneous T‐cell attracting chemokine), MIP1b (macrophage inflammatory protein 1b) and PDGFbb (platelet‐derived growth factor BB) were proven to relate causally to at least one sarcopenia and aging trait in both analyses with two significance thresholds. IL16 was causally associated with hand grip strength (0.977 [0.956–0.998] for EWGSOP and 0.933 [0.874–0.996] for FNIH), AALM (0.991 [0.984, 0.998]), MVPA (0.997 [0.995–1.000]) and AWCU10 (1.008 [1.003–1.013]). CTACK was proven to relate causally to hand grip strength (1.013 [1.007–1.019] for EWGSOP and 1.090 [1.041–1.142] for FNIH), AWCU10 (0.990 [0.986–0.994]) and telomere length (0.998 [0.983–0.994]). The results indicated that MIP1b has a causal effect on hand grip strength (1.032 [1.001–1.063] for EWGSOP), AWCU10 (0.994 [0.988–1.000] and 0.993 [0.988–0.998]) and telomere length (1.006 [1.000–1.012]). PDGFbb may causally relate to AALM (1.016 [1.001–1.030]) and telomere length (1.011 [1.007–1.015]). Reserve MR analyses also proved their unidirectional causal effects.

**Conclusions:**

Twenty‐seven factors were causally related to sarcopenia and aging traits, and the causal effects of IL16, CTACK, MIP1b and PDGFbb were proven in both analyses with two significance thresholds.

## Introduction

Sarcopenia, defined as the age‐related decline in skeletal muscle mass, strength and function, is a prevalent geriatric syndrome associated with numerous adverse health outcomes, such as falls, fractures, disability and even death.^1^ In recent years, with the aggravation of an aging society, sarcopenia has gradually become a major challenge for both public health and older patients.^2^ It is estimated that sarcopenia affects approximately 10–40% of individuals aged 60 years and older, with the prevalence increasing to over 50% in those over the age of 80.^3^

The aetiology of sarcopenia is multifactorial, involving a complex interplay of age‐related changes in hormonal, inflammatory and metabolic pathways, as well as lifestyle factors such as smoking, physical inactivity and poor nutrition.^4^ Chronic low‐grade inflammation, known as inflammaging, has been identified as a key risk factor for the development and progression of sarcopenia, promoting muscle protein breakdown and impairing muscle regeneration.^5^ In recent years, inflammaging has emerged as a key concept in the field of gerontology. It is now widely recognized that aging is associated with a state of increased pro‐inflammatory markers and dysregulated immune responses, which relate to the development and progression of age‐related diseases.^6^

Understanding the mechanisms and consequences of inflammaging is of great importance, as it offers potential targets for interventions aimed at preventing or attenuating age‐related diseases, including sarcopenia. While it is widely hypothesized that the extension of lifespan and maintenance of a healthy lifespan are likely the outcomes of a well‐regulated equilibrium between pro‐inflammatory and anti‐inflammatory responses, the causal associations of inflammaging with aging and sarcopenia remain controversial.^5^ Previous studies have indicated that cytokines and growth factors play a crucial role in inflammaging by regulating the pro‐inflammatory and anti‐inflammatory pathways.^7^, ^8^ Cytokines and growth factors may serve as a bridge in studying the relationship between inflammaging and sarcopenia, as well as aging traits.

Observational studies in human populations have reported associations between cytokines and sarcopenia: Patients with abnormal levels of cytokines may face a higher risk of sarcopenia.^9^ However, due to the nature of observational studies and the high cost of randomized controlled trials (RCTs), it is inconclusive whether changes in cytokines are a cause or a consequence of sarcopenia. In this study, we aim to explore the causal relationship between cytokines, growth factors and sarcopenia using bidirectional Mendelian randomization (MR) approaches. Five kinds of sarcopenia traits and telomere length as aging traits were included in our study as outcomes.^10^ MR is a powerful analytical approach that leverages single nucleotide polymorphisms (SNPs) as instrumental variables (IVs) to investigate causal relationships between modifiable exposures and outcomes in observational studies. In this study, we hypothesized that some cytokines and growth factors may have causal effects on sarcopenia. By synthesizing the available evidence from observational studies, MR analyses and transcriptomes, we hope to shed light on the potential causal roles of cytokines and growth factors in the pathogenesis of sarcopenia. Understanding the intricate interplay between cytokines, growth factors and sarcopenia may ultimately contribute to the development of targeted therapeutic interventions aimed at mitigating the progression of sarcopenia for older patients, as well as elucidate the intricate roles of inflammaging in sarcopenia and aging for researchers.

## Methods

### Study design

**Figure 1:**
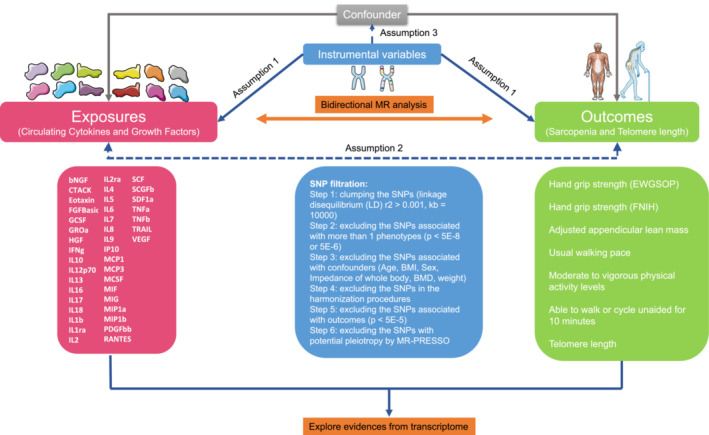
Overall design of our study. In this study, we aim to explore the causal association of cytokines with sarcopenia and aging traits and further identify the significant inflammation factors by using bidirectional Mendelian randomization. Forty‐one markers and seven sarcopenia and aging traits were included, and six single nucleotide polymorphism (SNP) filtration steps were employed to satisfy the three major assumptions for Mendelian randomization (MR) analysis. Inverse‐variance weighted, MR‐Egger and weighted median were employed to estimate the causality.

This study was an integrated study. The part of the bidirectional, two‐sample MR study was reported according to the STROBE‐MR. The overall design of our study is shown in *Figure* *1*. Our analysis used publicly available genome‐wide association study (GWAS) summary data and high‐throughput sequencing expression data. The data were acquired on 20 January 2023, and due to the nature of GWAS summary data, no personally identifiable information about individual participants was accessed during or after data collection. New ethics approval and consent to participate were not required. The ethics approval and consent to participate in each GWAS could be found in the raw studies.

Briefly, in the MR analyses, 41 kinds of circulation cytokines and growth factors were set as exposures, and 7 kinds of sarcopenia and aging traits were selected as outcomes. Telomere length was selected as an aging trait.^10^ The six kinds of sarcopenia traits were hand grip strength (European Working Group on Sarcopenia in Older People [EWGSOP]),^11^ hand grip strength (Foundation for the National Institutes of Health [FNIH]),^11^ adjusted appendicular lean mass (AALM),^12^ usual walking pace, moderate‐to‐vigorous physical activity (MVPA) levels^13^ and able to walk or cycle unaided for 10 min (AWCU10). Then the reversed MR was performed to identify the direction of the causal effects of circulation cytokines and growth factors. Then, to explore the expression of cytokines and growth factors in muscle tissues, analyses based on expression data were also conducted.

### Exposures

The detailed definition and information about 41 kinds of cytokines and growth factors can be found in *Table* *S1*. Study populations of cytokines and growth factors were from three cohorts (*Table* *S2*), including the Cardiovascular Risk in Young Finns Study (YFS; sample size = 1980, mean age = 37, mean body mass index [BMI] = 26.1 kg/m^2^), FINRISK1997 (sample size = 4608, mean age = 60, mean BMI = 27.9 kg/m^2^) and FINRISK2002 (sample size = 1705, mean age = 48, mean BMI = 26.6 kg/m^2^).^14^ Finally, 8293 individuals of European descent were included. The mean age of the population was 52, and the mean BMI was 27.2 kg/m^2^. The total follow‐up span was from 1980 to 2011. The cytokines were measured using Bio‐Rad's premixed Bio‐Plex Pro Human Cytokine 27‐plex assay and 21‐plex assay and a Bio‐Plex 200 reader with Bio‐Plex 6.0 software.^14^ Of the 48 measured cytokines, 7 were excluded due to the high rates of missing values (>90%).

### Outcomes

According to the sarcopenia (ICD‐10‐CM: M62.84) definition of the European Working Group on Sarcopenia in Older People 2 (EWGSOP2), sarcopenia was detected by using low muscle strength as the primary parameter and confirmed by the presence of low muscle quantity or quality.^1^ Severe sarcopenia is diagnosed when low muscle strength, low muscle quantity/quality and low physical performance are all detected. In this study, hand grip strength as a powerful measure was used as the parameter of muscle strength. AALM was measured as the parameter of muscle quantity, and MVPA, usual waking pace and AWCU10 were used as evaluations of physical performance.

The researchers in the raw cohorts measured and diagnosed the individuals. For the hand grip strength traits, two definitions of sarcopenia were used to determine the poor hand grip strength: the EWGSOP definition (male: <30 kg; female: <20 kg) and the FNIH definition (male: <26 kg; female: <16 kg).^11^ Datasets of hand grip strength contained 254 894 individuals aged 60 years or older from 22 cohorts, and the majority of populations were from the UK Biobank (*Table* *S2*).^11^ Various genotyping platforms were used in individual studies, and imputation was primarily carried out utilizing the Haplotype Reference Consortium (HRC) v1.1 panel. A total of 48 596 and 20 335 individuals were defined as having weak muscle strength based on EWGSOP and FNIH criteria, respectively. The other outcome cohorts were from UK Biobank cohorts. For the AALM cohort, the body composition was measured by the bioelectric impedance analysis (BIA) approach, and 450 243 individuals aged between 48 and 73 were finally included.^12^ The measurement of the usual walking pace, MVPA and AWCU10 is based on self‐report and wrist‐worn accelerometry data. A total of 459 915, 377 234 and 68 537 individuals were included in our study for those three traits, respectively.^13^ The telomere length was measured using a quantitative PCR assay, and 472 174 individuals were included.^15^ The study span for UK Biobank was from 2006 to 2010. The specific value assignment for each outcome can be found in *Table* *S2*.

### Datasets

Populations within the dataset used for the exposure and outcome variables are independent of each other, which met the basic assumption of a two‐sample MR study. The detailed information of the cohorts included in our study, including PubMed ID, database ID, sample size and so on, is summarized in *Table* *S2*.

High‐throughput gene expression data for 41 cytokines and growth factors were collected from the Gene Expression Omnibus (GEO) database (http://www.ncbi.nlm.nih.gov/geo/). The dataset ‘GSE111017’ containing expression data of muscle tissues from 119 individuals with or without sarcopenia was included in our study.

### Instrumental variable selection

First, the significance threshold of IVs associated with exposures was set as *P* < 5 × 10^−8^, but only 10 kinds of cytokines and growth factors had adequate numbers of IVs for further MR analyses. Therefore, at the threshold of *P* < 5 × 10^−6^, the MR was performed again to better prove the results. Then six strict IV selection steps were set to satisfy the three major assumptions for MR analysis and to reduce the bias (*Tables* *S3* and *S4*). In Step 1, a clumping procedure was implemented to ensure the absence of linkage disequilibrium (LD) (*r* ^2^ < 0.01, kb = 10 000). Subsequently, in Step 2, any IVs that were associated with more than one cytokine or growth factor were excluded (*Tables* *S5* and *S6*). In Step 3, to meet the assumption of independence, the IVs associated with confounders such as age, BMI, sex and weight were removed (*Table* *S7*) using PhenoScanner.^16^ In Steps 4 and 5, we eliminated the palindromic SNPs and the SNPs that showed an association with outcomes (*P* < 5 × 10^−5^). To address the potential influence of horizontal pleiotropy, Step 6 involved performing the MR‐PRESSO outlier test to calculate *P*‐values for each SNP and subsequently removing any SNPs identified as outliers.

### Main Mendelian randomization analyses

A bidirectional, two‐sample MR analysis was conducted to elucidate the causal relationships and directionality between cytokines, growth factors and sarcopenia. Three commonly used MR analysis methods, namely, inverse‐variance weighted (IVW) analysis, MR‐Egger regression and weighted median test, were employed. To address potential heterogeneity across studies, a random‐effects IVW analysis was employed, which yields more conservative estimates compared with the standard fixed‐effects IVW approach. All *P*‐values < 0.05 were considered statistically significant.

### Sensitivity analyses

Sensitivity analysis was performed to evaluate the strength of each IV in relation to the exposure of interest. The *F* statistics were calculated using the following formula: *F* = *R* ^2^ × (*N* − 2)/(1 − *R* ^2^), where *R* ^2^ was obtained using the following formula^4^: *R* ^2^ = [2 × BETA2 × EAF × (1 − EAF)]/[2 × BETA2 × EAF × (1 − EAF) + 2 × SE(BETA)2 × *N* × EAF × (1 − EAF)]. In this formula, BETA represents the genetic effects on exposures, EAF represents the effect allele frequency, SE(BETA) represents the standard error of the genetic effects and *N* represents the sample size. It is worth noting that all IVs included in our study had *F* statistics > 10. The statistical power of MR analysis was calculated using a web calculator (https://sb452.shinyapps.io/power/). To assess directional pleiotropy, we conducted the MR‐Egger intercept test. Cochran's Q test was employed to detect heterogeneity, with *P*‐values < 0.05 considered statistically significant for heterogeneity. To detect the characteristics and effects of single SNPs used in MR analysis, the forest plot, funnel plot and leave‐one‐out plot were visualized.

### Muscle tissue expression analyses

First, the Ensemble and Symbol IDs corresponding to the 41 cytokines and growth factors were converted. The expression matrix in GSE111017 was then searched for the corresponding genes. GSE111017 contains the expression profiling of muscle biopsies of the vastus lateralis muscle from 86 healthy individuals and 33 adults with sarcopenia. Gene expression differential analysis and visualization were performed for the 41 cytokines and growth factors between the normal group and the sarcopenia group. A *P*‐value < 0.05 was considered statistically significant for differential expression.

### Software

MR analyses were conducted using the ‘TwoSampleMR’ package Version 0.5.6 in R software Version 4.2.2 (R Foundation for Statistical Computing, Vienna, Austria). The MR analysis results were visualized using the R package ‘forestplot’ Version 3.1.1. The expression analyses were visualized using the R package ‘ggpubr’ Version 0.6.0.

## Results

### General characteristics

The cohorts for cytokines and growth factors included 8293 individuals, and for sarcopenia and aging traits, the sample sizes ranged from 69 537 to 472 174 (*Table* *S2*). The cohorts for cytokines and growth factors and those for sarcopenia and aging traits were independent of each other. Due to the different sets of significance thresholds of IVs associated with exposures, the main analyses and sensitivity analyses were performed two times according to different thresholds. The analyses with a threshold of *P* < 5 × 10^−8^ were defined as Analysis A, and those with a threshold of *P* < 5 × 10^−6^ were defined as Analysis B. After the steps for IV selection, 41 SNPs and 404 SNPs were finally included in the main analyses, respectively, for Analyses A and B. The detailed information on the final included SNPs is summarized in *Tables* *S8* and *S9*.

### Main Analysis A

**Figure 2:**
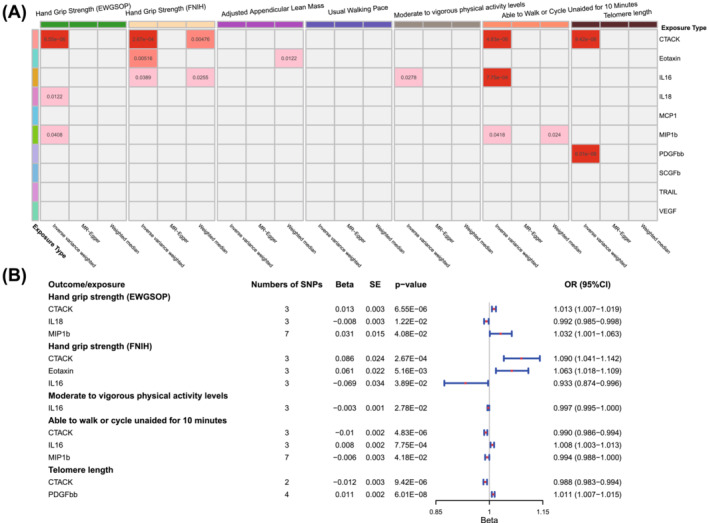
Results of Main Analysis A. (A) Heatmap of the causal associations of cytokines and growth factors with sarcopenia and aging traits. The grey cells represent no significant association, and the depth of the red cell means the *P*‐value. A deeper cell means a smaller *P*‐value. (B) Detailed information on the cytokines and growth factors with causal relationships to sarcopenia and aging traits.

Analysis A was conducted under the significance threshold of *P* < 5 × 10^−8^. The detailed results of Main Analysis A are summarized in *Table* *S10*. As shown in *Figure* *2* *A,B*, six cytokines and growth factors were identified to relate to the sarcopenia traits and telomere length: CTACK (cutaneous T‐cell attracting chemokine), eotaxin, IL16 (interleukin‐16), IL18 (interleukin‐18), MIP1b (macrophage inflammatory protein 1b) and PDGFbb (platelet‐derived growth factor BB). The estimated causal factors for hand grip strength (EWGSOP) were CTACK (95% confidence interval [CI]: 1.007–1.019, *P* = 6.55 × 10^−6^), IL18 (95% CI: 0.985–0.998, *P* = 0.012) and MIP1b (95% CI: 1.001–1.063, *P* = 0.041), and those for hand grip strength (FNIH) were CTACK (95% CI: 1.041–1.142, *P* = 2.67 × 10^−4^), eotaxin (95% CI: 1.018–1.109, *P* = 0.005) and IL16 (95% CI: 0.874–0.996, *P* = 0.039). For MVPA, only IL16 was estimated to have causal effects (95% CI: 0.995–1.000, *P* = 0.028). Moreover, CTACK (95% CI: 0.986–0.994, *P* = 4.83 × 10^−6^), IL16 (95% CI: 1.003–1.013, *P* = 7.75 × 10^−4^) and MIP1b (95% CI: 0.988–1.000, *P* = 0.042) were identified to relate to AWCU10 causally. For the aging traits, CTACK (95% CI: 0.983–0.994, *P* = 9.42 × 10^−6^) and PDGFbb (95% CI: 1.007–1.015, *P* = 6.01 × 10^−8^) were causally associated with telomere length.

The CTACK was estimated to causally relate to four sarcopenia traits: High serum levels of CTACK were causally associated with poor hand grip strength (both EWGSOP and FNIH), low ability to walk or cycle unaided for 10 min and poor telomere length. CTACK was proven to be a risk factor for sarcopenia in Analysis A. Similar to CTACK, MIP1b was also estimated to be associated with poor sarcopenia traits, including poor hand grip strength (EWGSOP) and AWCU10. The causal effects of IL16 were paradoxical: IL16 was proven to be a protective factor for hand grip strength (EWGSOP) and AWCU10, while being a risk factor for MVPA.

### Main Analysis B

**Figure 3:**
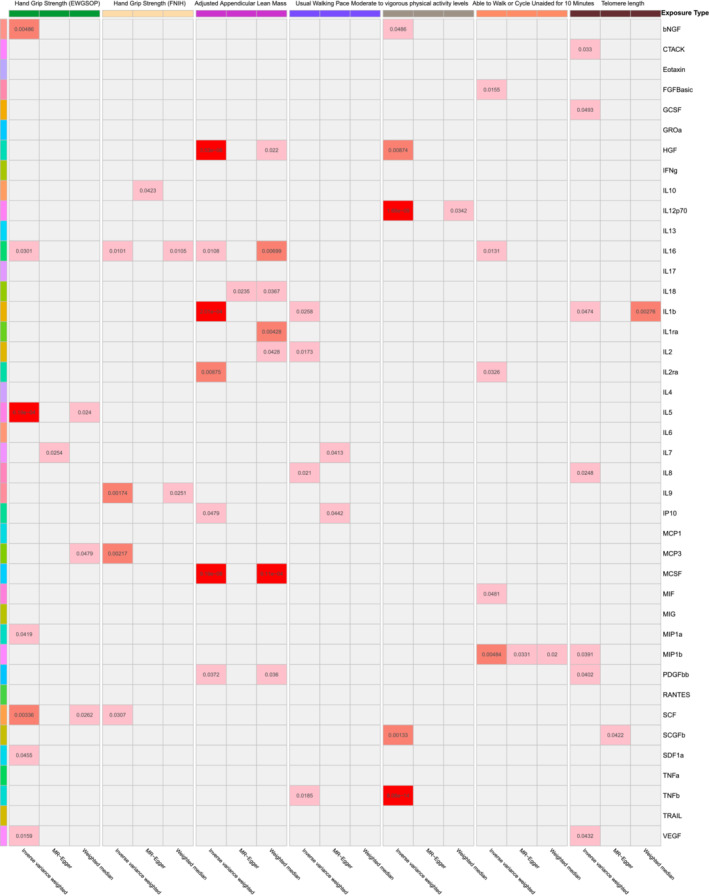
Heatmap of the causal associations of cytokines and growth factors with sarcopenia and aging traits in Analysis B. The grey cells represent no significant association, and the depth of the red cell means the *P*‐value. A deeper cell means a smaller *P*‐value.

**Figure 4:**
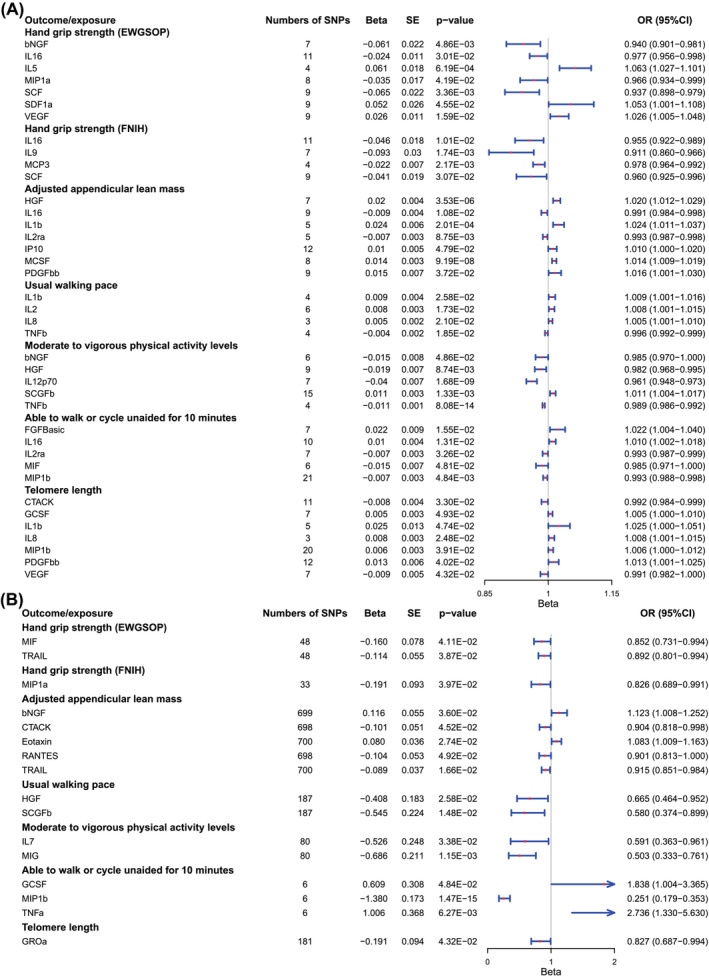
(A) Detailed information on the cytokines and growth factors with causal relationships to sarcopenia and aging traits in Analysis B. (B) Significant results in reverse analysis (sarcopenia as exposure and cytokines as outcome).

Analysis B was performed with a significance threshold of *P* < 5 × 10^−6^. The detailed results of Main Analysis B are summarized in *Table* *S11* and *Figure* *3*. Detailed information on the cytokines and growth factors with causal relationships to sarcopenia and aging traits is visualized in *Figure* *4* *A*. Compared with Main Analysis A, more cytokines and growth factors were identified to have causal effects on sarcopenia and aging traits. In the analyses for causal relationships between cytokines, growth factors and hand grip strength (EWGSOP), seven cytokines and growth factors with causal effects were identified: bNGF (beta nerve growth factor; 95% CI: 0.901–0.981, *P* = 0.005), IL16 (95% CI: 0.956–0.998, *P* = 0.030), IL5 (interleukin‐5; 95% CI: 1.027–1.101, *P* = 6.19 × 10^−4^), MIP1a (macrophage inflammatory protein 1a; 95% CI: 0.934–0.999, *P* = 0.042), SCF (stem cell factor; 95% CI: 0.898–0.979, *P* = 0.003), SDF1a (stromal‐cell‐derived factor 1 alpha; 95% CI: 1.001–1.108, *P* = 0.046) and VEGF (vascular endothelial growth factor; 95% CI: 1.005–1.048, *P* = 0.016). Similarly, IL16 (95% CI: 0.922–0.989, *P* = 0.010), IL9 (interleukin‐9; 95% CI: 0.860–0.966, *P* = 0.002), MCP3 (monocyte‐specific chemokine protein 3; 95% CI: 0.964–0.992, *P* = 0.002) and SCF (95% CI: 0.952–0.996, *P* = 0.031) were proven to relate to hand grip strength (FNIH) causally. For the causal relationships between cytokines, growth factors and AALM, seven factors were detected: HGF (hepatocyte growth factor; 95% CI: 1.012–1.029, *P* = 3.53 × 10^−6^), IL16 (95% CI: 0.984–0.998, *P* = 0.011), IL1b (interleukin‐1 beta; 95% CI: 1.011–1.037, *P* = 2.01 × 10^−4^), IL2ra (interleukin‐2 receptor alpha; 95% CI: 0.987–0.998, *P* = 0.009), IP10 (interferon gamma‐induced protein 10; 95% CI: 1.000–1.020, *P* = 0.048), MCSF (macrophage colony‐stimulating factor; 95% CI: 1.009–1.019, *P* = 9.19 × 10^−8^) and PDGFbb (95% CI: 1.001–1.030, *P* = 0.037). IL1b (95% CI: 1.001–1.016, *P* = 0.026), IL2 (interleukin‐2; 95% CI: 1.001–1.015, *P* = 0.017), IL8 (interleukin‐8; 95% CI: 1.001–1.010, *P* = 0.021) and TNFb (tumour necrosis factor beta; 95% CI: 0.992–0.999, *P* = 0.019) were proven to be causally associated with usual walking pace. Moreover, five cytokines and growth factors with causal effects on MVPA were identified: bNGF (95% CI: 0.970–1.000, *P* = 0.049), HGF (95% CI: 0.968–0.995, *P* = 0.009), IL12p70 (interleukin‐12p70; 95% CI: 0.948–0.973, *P* = 1.68 × 10^−9^), SCGFb (stem cell growth factor beta; 95% CI: 1.004–1.017, *P* = 0.001) and TNFb (95% CI: 0.986–0.992, *P* = 8.08 × 10^−14^). For the AWCU10 trait, FGFBasic (fibroblast growth factor basic; 95% CI: 1.004–1.040, *P* = 0.016), IL16 (95% CI: 1.002–1.018, *P* = 0.013), IL2ra (95% CI: 0.987–0.999, *P* = 0.033), MIF (macrophage migration inhibitory factor; 95% CI: 0.971–1.000, *P* = 0.048) and MIP1b (95% CI: 0.988–0.998, *P* = 0.005) may have a significantly causal effect. Seven causal associations of cytokines and growth factor with telomere length were identified: CTACK (95% CI: 0.984–0.999, *P* = 0.033), GCSF (granulocyte colony‐stimulating factor; 95% CI: 1.000–1.010, *P* = 0.049), IL1b (95% CI: 1.000–1.051, *P* = 0.047), IL8 (95% CI: 1.000–1.015, *P* = 0.025), MIP1b (95% CI: 1.000–1.012, *P* = 0.039), PDGFbb (95% CI: 1.000–1.025, *P* = 0.040) and VEGF (95% CI: 0.982–1.000, *P* = 0.043).

In summary, IL16 had the largest number of causal associations with sarcopenia and aging traits, but the results were still paradoxical. IL16 may have positive effects on hand grip strength and AWCU10 while having negative effects on AALM. Moreover, the results indicated that PDGFbb was a protective factor for AALM and telomere length, and the positive effects of PDGFbb were also suggested in Analysis A. Similar to the results of Analysis A, high levels of CTACK were causally associated with poor telomere length, and MIP1b was also proven to be the risk factor for AWCU10.

### Sensitivity analyses

The *R* ^2^ and *F* statistics of IVs in relation to cytokines and growth factors in Analyses A and B and the statistical power of each MR analysis are summarized in *Tables* *S12* and *S13*. All *F* statistics were strong enough for MR analysis (>10). Directional pleiotropy was evaluated by using the MR‐Egger intercept test, and heterogeneity was detected by using Cochran's Q tests. The results of the MR‐Egger intercept test and Cochran's Q tests in Analyses A and B are summarized in *Tables* *S14* and *S15*. In the sensitivity analyses for Analysis A, except for the estimates of TRAIL (tumour necrosis factor [TNF]‐related apoptosis‐inducing ligand) on AALM (*P* = 0.039), no directional pleiotropy was detected. In Cochran's Q test, most estimates were not proven to have heterogeneity (*Table* *S14*). In the sensitivity analyses for Analysis B, directional pleiotropy was found in three relationships: IL7 (interleukin‐7) and hand grip strength (EWGSOP) (*P* = 0.046), IL10 (interleukin‐10) and hand grip strength (FNIH) (*P* = 0.048), and IL7 and usual walking pace (*P* = 0.025). Similar to Analysis A, in Cochran's Q test for Analysis B, most estimated associations may not have heterogeneity (*Table* *S15*). The forest plot, funnel plot and leave‐one‐out plot for SNPs in both forward and reverse analyses can be found in the supporting information data (*Figures* *S1*–*S644*).

### Reverse analyses

To identify the directionality of the causal associations, reverse analyses as the second step of bidirectional MR analyses were performed. The six sarcopenia traits and telomere length were set as exposure traits, while the cytokines and growth factors were selected as outcomes. The detailed results of reverse main analyses are summarized in *Table* *S16*, and the results of reverse sensitivity analyses are summarized in *Tables* *S17* and *S18*. Though a few factors were identified to have reverse causality to sarcopenia and aging traits (*Figure* *4* *B*), almost all these factors were not proven to have causal effects in Analyses A and B, except MIP1b (95% CI: 0.179–0.353, *P* = 1.47 × 10^−15^ in reverse analyses, with AWCU10). In brief, except for the relationships between MIP1b and AWCU10, all the cytokines and growth factors were causally associated with sarcopenia and aging traits unidirectionally.

### Muscle tissue expression analyses

**Figure 5:**
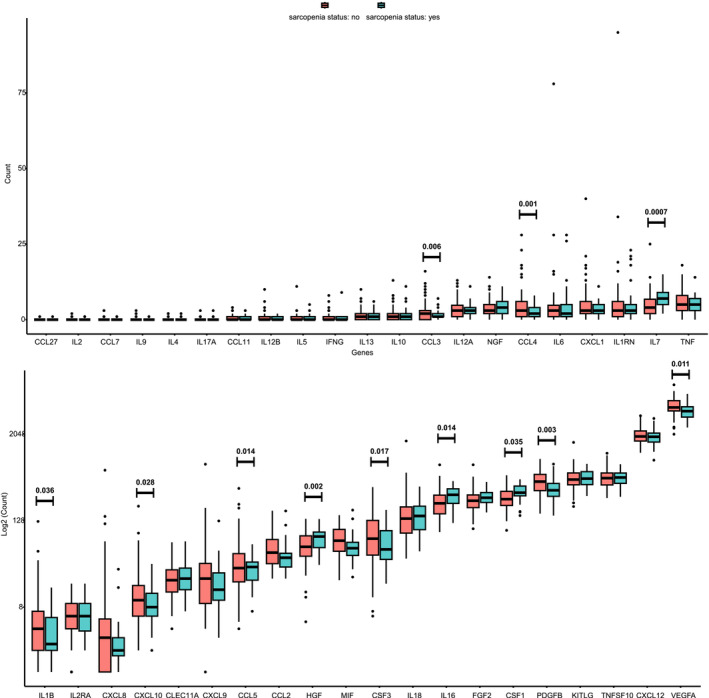
Comparison of expression levels of genes in relation to cytokines and growth factors in muscle tissue (normal individuals vs. individuals with sarcopenia).

The raw expression matrix of muscle tissue from both individuals without and with sarcopenia and the basic information and identifications of cytokines and growth factors are summarized in *Table* *S19*. The expression levels of genes corresponding to cytokines and growth factors in normal populations were compared with those in sarcopenia populations, and the results are summarized in *Table* *S20*. The different expression levels of 12 cytokines and growth factors in muscle tissues from individuals with and without sarcopenia were identified (*Figure* *5*).

## Discussion

Inflammaging, characterized by increased levels of pro‐inflammatory cytokines and altered immune cell function, is a prominent feature of aging. It relates to the development of sarcopenia, a multifactorial condition involving muscle wasting, decreased muscle strength and impaired muscle regeneration.^5^ Due to the impact of the overall body environment in older age, understanding the changes in the overall body inflammatory environment may provide new insights into the complex mechanisms of sarcopenia. Cell signalling factors, such as cytokines and growth factors, play crucial roles in the regulation of inflammaging,^17^ and cytokines and growth factors in serum may also reveal the comprehensive and overall body inflammaging status.

Previous population studies have proven the relationships between cytokines, growth factors, sarcopenia and aging.^18^ The relationship between IL6 (interleukin‐6) and sarcopenia in individuals aged more than 65 (*P* = 0.0001) and the relationship between TNFa (tumour necrosis factor alpha) and sarcopenia in all individuals (*P* = 0.020) have been reported in a meta‐analysis including 80 studies.^19^ Moreover, after reviewing the studies focusing on the relationships between cytokines, growth factors and sarcopenia, many factors, including IL1b, IL6, IL8, IL17 (interleukin‐17), TNFa, TGF1, GDF‐15 (growth differentiation factor‐15) and so on, may all relate to sarcopenia.^18^, ^20^, ^21^, ^22^

**Figure 6:**
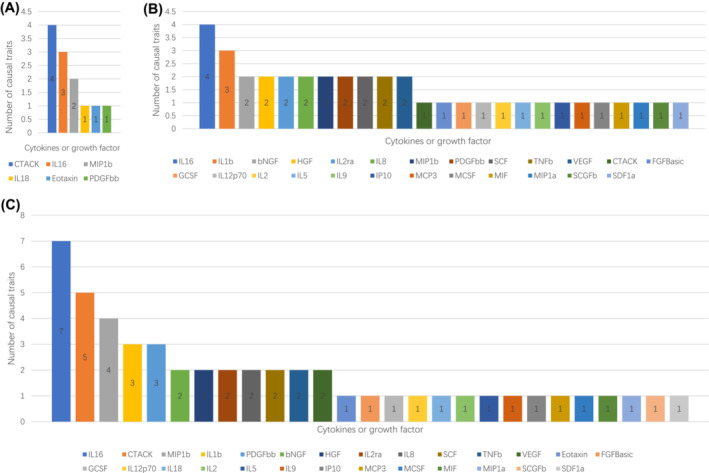
Numbers of sarcopenia traits related to cytokines and growth factors causally. (A) In Analysis A only. (B) In Analysis B only. (C) Sum numbers in Analyses A and B.

Though the relationships between cytokines, growth factors and sarcopenia traits could be identified by observational study, the causal associations between them were still controversial. In this study, by using the MR method, we identified 27 cytokines and growth factors that may relate to sarcopenia and aging traits causally. Most of these factors were associated with only one sarcopenia and aging trait (*Figure* *6*). To identify the significance of the causal effects of cytokines and growth factors on sarcopenia, we calculated the number of causal traits for each factor and visualized them in *Figure* *6*. IL16, CTACK, MIP1b and PDGFbb were proven to relate to at least one sarcopenia and aging trait causally in both Analyses A and B. Reserve MR analyses also proved their unidirectional causal effects. Therefore, we estimated that these four factors may have the most significant evidence for their causal effects on sarcopenia and aging traits.

IL16 is a pro‐inflammatory cytokine that plays a crucial role in various physiological and pathological processes. It is primarily produced by activated T lymphocytes and serves as a chemoattractant for various immune cells, including CD4+ T cells, monocytes and dendritic cells.^23^ IL16 exerts its effects through binding to CD4 and acts as a potent modulator of immune responses by promoting T cell activation, recruitment and proliferation.^24^ IL16 may lead to the release of TNF, IL1 (interleukin‐1), IL6, IL15 (interleukin‐15) and IL12 (interleukin‐12) by activating CD4 T cells,^25^ and these cytokines may contribute to the transformation of the pro‐inflammatory environment. IL16 has been implicated in several inflammatory diseases, including asthma, rheumatoid arthritis, inflammatory bowel disease and systemic sclerosis.^26^ In the results of MR analyses, the causal roles of IL16 in different sarcopenia traits were controversial: IL16 may have positive effects on hand grip strength and AWCU10 while having negative effects on AALM and MVPA. This phenomenon was usually due to the bias caused by the selection of specific SNPs; however, the sensitivity analysis indicated that this bias was not significant. Therefore, we have to consider that IL16 may have different effects on sarcopenia traits. Previous population studies have reported that IL16 has a sex‐specific association with sarcopenia: IL16 may be a protective factor for males and a risk factor for females.^9^ Similarly, in individuals with gastric cancer, patients with higher levels of IL16 may have a higher risk of sarcopenia.^27^ In the expression analyses of muscle tissues, the patients with sarcopenia also have higher levels of IL16 expression (*Figure* *5*). However, little evidence from cellular and molecular levels was reported, and the precise mechanisms by which IL16 contributes to the development of sarcopenia remain unclear. The effects of IL16 on sarcopenia need more experimental evidence.

CTACK, also known as CCL27, is a chemokine that belongs to the CC chemokine family. It is primarily expressed by keratinocytes, which are the predominant cell type in the epidermis and are involved in various physiological and pathological processes.^28^ CTACK exerts its effects by binding to CCR10, which is predominantly expressed in T cells and dendritic cells. CTACK–CCR10 signalling is involved in the regulation of immune responses, including the recruitment of T cells to the skin during inflammation and the formation of immunological synapses between dendritic cells and T cells.^29^ CCR10/CTACK interaction may relate to the development of sarcopenia due to their crucial roles in regulating multiple immune cells with conflicting effects, including helper T cells, cytotoxic T cells, regulatory T cells, natural killer cells, myeloid‐derived suppressor cells and so on.^30^ However, few studies have reported the relationships between CTACK and sarcopenia. More research is needed to elucidate the relationships.

Similar to CTACK, MIP1b is also a chemokine that belongs to the CC chemokine family. MIP1b exerts its effects by binding to CCR5 and CCR1.^31^ MIP1b‐mediated recruitment and activation of immune cells are essential for the initiation and maintenance of immune responses, as well as the resolution of inflammation.^32^ The precise mechanisms underlying the involvement of MIP1b in the pathogenesis of muscular dystrophy remain largely elusive, and there is limited literature available on this topic. One possible mechanism by which MIP1b may contribute to sarcopenia is through the recruitment and activation of immune cells, such as macrophages and T cells, in affected muscle tissues. MIP1b may also play a role in sarcopenia by binding to CCR5, as CCR5 is expressed in muscle cells, and inhibiting CCR5 may block the myogenesis stimulated by CCL11.^33^

PDGFbb is a potent mitogenic factor and plays a crucial role in various biological processes, including cell proliferation, migration and tissue repair.^34^ PDGFbb is primarily secreted by platelets, macrophages and endothelial cells in response to tissue injury or inflammation. In a population study conducted in Poland, the older males enrolled in this study had a significantly lower PDGFbb level compared with young men.^35^ In our study, PDGFbb was also proven to be a positive causal factor for longer telomere length and higher AALM. PDGFbb may promote the migration and proliferation of dermal fibroblasts and then improve injury healing and cell growth.^36^ Another possible explanation for the relationships is that PDGFbb may play a protective role when facing chronic injuries or inflammation in muscle tissues, so individuals with higher levels of PDGFbb may have a stronger resistance to sarcopenia. PDGFbb may also affect the development of sarcopenia by regulating bone regeneration and leading to aging metabolic stress traits.^37^ Moreover, some studies have reported that PDGFbb may regulate myogenic proliferation and differentiation by interacting with satellite cells.^38^

This is the first MR analysis to explore the causal roles of cytokines and growth factors in sarcopenia and aging traits. After a comprehensive analysis of the MR results, IL16, CTACK, MIP1b and PDGFbb were identified as the factors with the most significant causal effects. However, the limitations should be taken into account when interpreting the results. First, as strict IV filtration steps were set to satisfy the basic assumptions of MR analyses, the number of IVs may be limited. Second, the individuals in our study were of European descent. Different populations may have varying genetic backgrounds and environmental factors that influence the relationship between cytokines, sarcopenia and aging traits. Third, in tests for some factors' causal effects, directional pleiotropy was detected, and the bias should be considered when discussing these results. However, the causal relationships were not proven to be significant and were not discussed, so the bias may not affect the overall conclusion. Next, some MR analyses in our study may have heterogeneity; however, the random‐effects IVW model as a more conservative model may not be affected by heterogeneity significantly.^39^ Moreover, the GWASs of exposures and outcomes may be from different periods, and this may cause bias in estimating the causal associations. However, MR analysis identifies the causal relationships based on the assumption of random assortment in the genetic make‐up formation, and this bias may not be significant in MR analysis. Lastly, instead of serum levels, expression analyses of muscle tissue may provide insights for specific tissue expression of cytokines and growth factors, and the expression levels in muscle tissue may not present protein levels, so the results of expression analyses are only of reference value. And the expression levels of factors in muscle tissue may not present the protein levels.

In conclusion, our study identified potential causal relationships between certain cytokines and these age‐related conditions. The causal effects of IL16, CTACK, MIP1b and PDGFbb were proven in both analyses with two significance thresholds, which suggested that these four factors may have the most significant causal effects on sarcopenia and aging traits. Future research should focus on further elucidating the underlying mechanisms and exploring the potential of targeting cytokines as a therapeutic strategy for sarcopenia and aging‐related conditions. This may ultimately lead to the development of novel interventions to improve the health and quality of life of aging populations.

## Funding

This work was supported by the National Natural Science Foundation of China (Grant/Award Number: 81971169) and the Leading Talents Training Program of Pudong New Area Health Commission (Grant ID: PWR 12020‐06).

## Conflict of interest statement

The authors declare that they have no conflict of interest.

## Supporting information


**Data S1.** Supporting Information

**Table S1:** Detailed definition and information about the circulation cytokines and growth factors
**Table S2:** Detailed information about cohorts of exposures and outcomes
**Table S3:** IVs selection steps in the threshold of *p* < 5E‐08
**Table S4:** IVs selection steps in the threshold of *p* < 5E‐06
**Table S5:** IVs that were associated with more than one cytokine or growth factor in the threshold of *p* < 5E‐08
**Table S6:** IVs that were associated with more than one cytokine or growth factor in the threshold of *p* < 5E‐06
**Table S7:** IVs that were associated with confounders in the threshold of *p* < 5E‐06
**Table S8:** Final included SNPs in the threshold of *p* < 5E‐08
**Table S9:** Final included SNPs in the threshold of *p* < 5E‐06
**Table S10:** Results of main analyses in the threshold of *p* < 5E‐08
**Table S11:** Results of main analyses in the threshold of *p* < 5E‐06
**Table S12:** R^2^ and F statistics of IVs in relation to cytokines and growth factors in analyses A
**Table S13:** R^2^ and F statistics of IVs in relation to cytokines and growth factors in analyses B.
**Table S14:** Results of sensitivity analyses of analyses A.
**Table S15:** Results of sensitivity analyses of analyses A.
**Table S16:** Results of reverse main analyses.
**Table S17:** R^2^ (%) and F statistics of reverse main analyses.
**Table S18:** Results of reverse sensitivity analyses.
**Table S19:** Expression matrix.
**Table S20:** Results of differential expression analyses.

## Data Availability

The UK Biobank data can be found at http://www.nealelab.is/uk‐biobank/. Data from meta‐GWASs can be found in the raw publications. The PubMed ID or the database ID of each GWAS can be found in *Table* *S1*. The data generated in our study can be found in the supporting information tables. The supporting information figures can be accessed through the following links: https://www.alipan.com/s/k1wTxwF7ogy.
